# Building community and public engagement in research – the experience of early career researchers in East Africa

**DOI:** 10.12688/aasopenres.13349.1

**Published:** 2022-03-16

**Authors:** Joel L. Bargul, Denna M. Mkwashapi, Imelda Namagembe, Immaculate Nakityo, Annettee Nakimuli, Josaphat Byamugisha, Daniel Semakula, Janet Seeley, Nelson K. Sewankambo

**Affiliations:** 1 Animal Health Theme, International Centre of Insect Physiology and Ecology (icipe), Nairobi, P.O. Box 30772-00100, Kenya; 2 Department of Biochemistry, Jomo Kenyatta University of Agriculture and Technology (JKUAT), Nairobi, P.O. Box 62000-00200, Kenya; 3 THRiVE, c/o School of Medicine, Makerere University College of Health Sciences, Kampala, P.O. Box 7072, Uganda; 4 National Institutes for Medical Research, Mwanza, P.O. Box 1462, Tanzania; 5 School of Medicine, Makerere University College of Health Sciences, Kampala, P.O. Box 7072, Uganda; 6 Mulago Specialized Women and Neonatal Hospital (MSWNH), Kampala, P.O Box 7272, Uganda; 7 Department of Global Health and Development, London School of Hygiene and Tropical Medicine, London, WC1H 9SH, UK

**Keywords:** Public engagement, health research, Kenya, Tanzania, Uganda, East Africa

## Abstract

**Background:** In this paper, we explain how three early career researchers actively engaged community members in health research in Kenya, Tanzania and Uganda in their research projects, and what was learnt from the experience. The research project in Kenya was on camel trypanosomiasis and the role of camel biting keds (or louse flies) in disease transmission. The project in Tanzania looked at the effect of human immunodeficiency virus (HIV) and antiretroviral therapy on fertility and ascertained the trends in the use of family planning services amongst women of reproductive age. The focus of the project in Uganda was the implementation of maternal death surveillance and the response policy to determine the cause of maternal deaths and how they might be prevented.

**Methods:** In the three different settings, efforts to ensure local community engagement provided a focus for the researchers to hone their skills in explaining research concepts and working in partnership with community members to co-develop ideas, their research methods and outputs.

**Results: **Involvement of communities in scientific research, which entailed a two-way mutual engagement process, led to (i) generation of new research ideas that shaped the work, (ii) strengthened mutual trust, and (iii) promoted uptake of research findings.

**Conclusions:** Our key findings strongly support the need for considering community engagement as one of the key components in research studies.

## Disclaimer

The views expressed in this article are those of the author(s). Publication in AAS Open Research does not imply endorsement by the AAS.

## Introduction

Over the past decade, there has been a growing awareness of the value of public engagement in research. The coronavirus disease 2019 (COVID-19) pandemic has highlighted how essential the public understanding of science can be to the acceptance of measures to curtail the spread of infection, with disinformation, often through social media, needing to be countered by authoritative and clearly expressed messages from researchers, epidemiologists, virologists and behavioural scientists throughout the world ( Larson, 2020; Plohl & Musil, 2021; Provenzi & Barello, 2020). For many scientists engaged in the rapidly evolving fields of COVID-19 research, the need to respond to media requests for information or explain their findings to particular interest groups, has become an important role in the effort to counter the spread of infection and encourage vaccine uptake ( Safford *et al.*, 2021; Umviligihozo *et al.*, 2020). The societal value placed on funding, doing and sharing the outputs of scientific research is nurtured through effective community and public engagement ( Holzer *et al.*, 2014)

Many different terms have been used to describe the involvement, the engagement and the participation of people from the community in which research takes place, and the wider public. Involving local community members in research can improve the relevance and quality of research, as those directly affected by the subject under study, for example an infectious disease, can draw attention to factors in the local environment which can enhance the usefulness of the research ( Tindana *et al.*, 2007).

We draw on the definition of public engagement used by Cohen *et al.* (2008: 2): ‘a process that provides people with trustworthy information on key policy issues, elicits their input, and integrates it into decision-making and social action’. They make a distinction between this broader engagement agenda and that of ‘community engagement’, where the people directly participating in or affected by a research project are the focus of engagement. Both are important, and we would argue that as an introduction to broader ‘public engagement’, local community engagement provides a focus for emerging scientists, for example, to hone their skills in explaining research concepts and to work with community members to develop ideas, methods and outputs.

In addition, there is an increasing awareness by research funders of the importance of community and wider public engagement, and for researchers to include a public engagement component in grant applications. The case is made in grant calls for public involvement in research to serve ‘broader democratic principles of citizenship, accountability and transparency’ ( National Institutes of Health Research, 2021) and increase public trust ( African Academy of Sciences, 2021). This increasing pressure to include a public engagement component in research projects can seem particularly daunting to early career researchers, embarking on an independent research project for the first time. Guidance exists, in an effort to demystify the terminology and encourage greater involvement in research as well as good practice ( African Academy of Sciences, 2021; Wellcome Trust, 2021),

In this paper we explain how three early career researchers actively engaged community members in research in Kenya, Tanzania and Uganda, and what we learnt from the experience.

Our research was supported through the Training Health Researchers into Vocational Excellence in East Africa ( THRiVE) project which is a collaborative research capacity building project involving five universities (Makerere University - Uganda, Gulu University - Uganda, Kilimanjaro Christian Medical University College - Tanzania, University of Cambridge – United Kingdom, UK, London School of Hygiene and Tropical Medicine - UK); and three research institutes (Uganda Virus Research Institute, International Centre of Insect Physiology and Ecology in Kenya and the National Institute of Medical Research, in Mwanza - Tanzania). The goal of THRiVE was to develop a critical mass of world class researchers and research leaders capable of conducting high quality independent research and transforming communities where they live and work. 

Our approach has been to build a cohort of researchers, providing support and mentoring to scientists at different stages of their research careers, including graduate interns, Masters, doctoral and post-doctoral researchers. We have focused on achieving research excellence in the areas of (a) infectious diseases/neglected tropical diseases, (b) maternal, neonatal and reproductive health, and (c) non-communicable diseases. In total, 70 research fellows have benefitted from supervision, teaching and mentoring in the project across the three East African countries.

We begin by describing the research settings, and the scientific research focus of our three case studies – research led by Joel, Denna, and Imelda, before recounting the public engagement activities undertaken, and our learning from that experience.

## Ethical approval

Joel’s study was undertaken in strict adherence to experimental guidelines and procedures approved by the *icipe* Institutional Animal Care and Use Committee, IACUC (REF: IACUC/ICIPE/003/2018), and the Pwani University Ethics Review (REF: ERC/EXT/002/2020). Animals were handled carefully to minimize pain and discomfort during sampling. Permission to engage with pastoralist farmers in research and to sample their livestock was obtained through verbal consent, as most herders were unable to read or write. Engagement of high school students in research was conducted after obtaining the permission from Laisamis Secondary School’s principal.

Denna’s project targeting women of reproductive age in Tanzania to determine the influence of human immunodeficiency virus (HIV) and antiretroviral therapy (ART) on fertility and the uptake of family planning services received ethical approval from the Review Committee of Kilimanjaro Christian Medical College of the Tumaini University of Tanzania (certificate number 2440). Permission to work with secondary school students was sought and obtained from the director, Mwanza city council (ref. No MCC/SE/20.VOL.II/127). Similarly, Imelda’s study received approval from the Makerere University School of Medicine Higher Degrees Research and Ethics Committee (SOMREC; #REC Ref 2018-001). In addition, the study was approved by the Uganda National Council for Science and Technology (UNCST; Ref SS4797) to conduct surveillance on maternal deaths and establish their causes. The study engaged with high school students in research, and this was preceded by a request for permission from the headmaster and director of studies to meet the students in the school.

### The settings and the background to the research project

***Camel trypanosomiasis and its transmission in northern Kenya*.** Joel Bargul’s research project in Kenya was on camel trypanosomiasis and the role of camel biting keds (or louse flies, genus *Hippobosca*) in disease transmission. The study was conducted in Laisamis, Marsabit County, about 450 km northeast of Nairobi City. The major economic activity in this vast arid and semi-arid region of northern Kenya is livestock keeping. Livestock are often the sole source of nutrition and livelihoods among the largely nomadic and pastoral communities. Camels are preferred to other livestock in many communities because of their resilience to survive in harsh climates with prolonged droughts downstream of global warming. They are kept for milk, meat, hides, transport, income, and for social capital. However, camel productivity is constrained by ectoparasites, biting flies, and the diseases they transmit. In northern Kenya, little information is available about diseases circulating in livestock or their possible transmission by hematophagous keds, the predominant ectoparasites that infest all camel herds all year round ( Bargul *et al.*, 2021). Keds also occasionally feed on humans and in the process, they could transmit zoonotic pathogens. Camels may harbour zoonotic pathogens including MERS-Coronavirus, Rift Valley Fever virus, *Coxiella*, *Bartonella*, *Anaplasma*, among others ( Zhu *et al.*, 2019). About 75% of newly emerging diseases currently affecting humans originated in animals ( Jones *et al.*, 2008).

Earlier findings from research in this area shows that African trypanosomiasis is one of the major camel diseases in Laisamis ( Kidambasi *et al.*, 2020), and tsetse fly (genus *Glossina*) is presently the only known definitive biological vector of this disease. However, tsetse flies are not found in this study region, yet the disease is widespread, so Joel led a project to investigate whether camel keds that belong to the same superfamily *Hippoboscoidae* as tsetse could spread trypanosomiasis (among other pathogens).

Therefore, the aim of this study Joel led as a part of THRiVE postdoctoral research fellowship was to determine disease transmission patterns among co-herded livestock and study the role of keds in their spread. Field sampling was done in Laisamis located in the south of Marsabit County. All necessary techniques, access to livestock herds in northern Kenya, and ethical permissions were already in place. Keds were collected from identity tagged animals and at the same time blood was collected through a cross-sectional study design by convenient sampling approach.

Camel keds were collected from camel herds and preserved in absolute ethanol and transported to the *icipe* laboratories for species identification and fly sorting by sex. Keds were also randomly collected from co-herded animals such as donkeys, cattle, goats, dogs, and sheep for morphological and molecular identification to establish whether camel keds could infest other livestock species.

By following published protocols with minor modifications on ked feeding assays ( Oyieke & Reid, 2003), Joel and his team studied the ability of camel keds to transmit blood-borne pathogens from naturally infected camels to experimental mice and rabbits through three independent repeat experiments. Thus, laboratory animals were transported to the field sampling sites in northern Kenya to provide bloodmeals for freshly collected camels keds and thereafter the samples from those laboratory mice and rabbits were used to identify transmitted ked-borne pathogens using molecular assays at *icipe* (Nairobi).

The findings of this study show that *Anaplasma* and *Ehrlichia* spp. and trypanosomes species *Trypanosoma vivax* and *T. evansi* are present in camels and in keds collected from them, suggesting a possible role in disease transmission ( Kidambasi *et al.*, 2020). Further, this study demonstrated, for the first time, that camel keds could transmit ‘ *Candidatus* Anaplasma camelii’ from camels to mice and rabbits via blood-feeding bites ( Bargul *et al.*, 2021). This camel *Anaplasma* pathogen that is poorly understood at present (i.e. infection mechanism, veterinary and zoonotic importance) was found in 63 – 78% of 976 camels sampled over a period of four years, from 2017 – 2020.

***Influence of HIV and antiretroviral therapy on fertility and uptake of family planning services*.** The project which Denna Michael led for his doctoral studies aimed to understand the effect of HIV and antiretroviral therapy (ART) on fertility and ascertain the trends in the use of family planning services amongst women of reproductive age in Tanzania.

With the changing HIV epidemic to a chronic illness and increased access to ART in Tanzania, it is not clear on how HIV has impacted on the fertility gap between HIV infected and uninfected women at different periods of ART coverage. Denna and his team wanted to know to what extent uptake of family planning services has been changing with time. For the study, Denna used data from the 25 year old - Magu Health and Demographic Surveillance System (HDSS) ( Kishamawe *et al.*, 2015) *.* The study area lies 20 km east of Mwanza City, the region's capital, with a predominantly rural population. Magu HDSS is comprised of nine villages, with a combined population of 45,000 people (by 2020).

The fertility data Denna used were drawn from 35 rounds of household visits from 1994 to 2018, which captured all births in the resident population. HIV status data were drawn from eight rounds of HIV epidemiologic and serologic surveillance, which was conducted every three years, from 1994 to 2018 among all eligible, resident adults aged 15 years and above. Using those data, Total fertility rate (TFR), Age specific fertility rate (ASFR), General fertility rate (GFR), Contraceptive use and Unmet need for contraception by HIV status and different levels of ART availability were compared over time and factors associated with the changes were investigated.

***Implementation of maternal death surveillance and response policy: the impact of training and community engagement*.** Imelda Namagembe research project in Uganda was based in the Department of Obstetrics and Gynaecology at Mulago National Referral and Teaching Hospital for the Makerere University located about 3 km northern part of Kampala (the capital city of Uganda). The hospital has one of the busiest labour wards in Africa with 39,000 deliveries a year ( Hughes *et al.*, 2020). The labour ward has about 46 obstetricians and gynaecologists ( Namwaya *et al.*, 2020). Over the past year two obstetricians have been on duty each day and one for the night cover to ensure quality health care from their specialist expertise. Health workers provide Reproductive Maternal Newborn, Child and Adolescent Health Services (RMNCAH) to all women attending the facility from all over Uganda. The National Referral Hospital is faced with a high burden of maternal and perinatal deaths and contributes the highest number of deaths to Kampala district within Uganda ( Ministry of Health, 2019). This is partly due the hospital being a referral site and most of the deaths are due to conditions that are preventable. Such as excessive bleeding, hypertensive disorders of pregnancy (pre-eclampsia/eclampsia), sepsis (bacterial infection) from obstructed labour and abortion related complications compounded by delay in seeking care, delay in getting transport and delays at health facilities ( Kiondo *et al.*, 2021; Nakimuli *et al.*, 2016). Most of the women who die, die young, with a mean age of about 26 years, and of these 10 to 15% are adolescents/young adults (< 20 years) ( Ministry of Health, 2018). One of the strategies to improve maternal and new-born outcomes is to conduct timely Maternal Death Surveillance and Response (MDSR) policy where the hospital staff are expected to notify a maternal death within 24 hours, conduct a death audit to identify gaps in care that contributed to the death within 7 days, develop recommendations and follow up implementation to prevent future deaths. However, this was not being done on a regular basis.

The aim of the study conducted by Imelda as part of THRiVE-funded research was to examine the implementation of maternal death surveillance and response policy for a 3-year period (2016 – 2018) as a baseline, determine cause of maternal deaths and preventability; this was followed by exploration of the barriers and facilitators to MDSR implementation and later evaluate the impact of training with stakeholder engagement on MDSR performance.

In the course of her research Imelda has interacted with a number of stakeholders in the hospital and externally (doctors, midwives and nurses, health workers, lawyers, and the laboratory and pharmacy support teams) to understand the barriers to quality improvement process of MDSR and what should be done in order to improve the outcomes for mothers and their babies.

All three research projects described above were conducted in community settings – albeit in three very different places: arid northern Kenya, rural north western Tanzania and the main referral hospital in the capital of Uganda. In the next section, we explain where our idea for the community engagement activities in the projects came from.

### Where did the idea for the community and public engagement (CPE) focus come from?

Joel’s work was firmly embedded in the day-to-day processes of camel herding, so the goodwill of the herders was critical to the success of the project. Joel had ensured that the key stakeholders, particularly the camel farmers, were familiar with the focus of the research and encouraged the exchange of ideas. The farmers used to freely discuss the key challenges they faced during livestock production, ranging from animal husbandry practices, the burden of pests and diseases, the transmission of diseases, and the traditional and modern ways used for control. Joel knew the work that he and his colleagues conducted would – if successful – help to address these pest and disease challenges. 

It was another area of daily life that Joel chose to focus his community engagement activities. During field visits to collect samples, the team commonly observed that children of school-going age were not enrolled in school, but engaged in other duties at home, such as livestock herding activities. Joel and his colleagues wanted to understand more about the value placed on education for children and the contribution of children to labour (including camel herding) with a view to trying to support great opportunities for the children to go to school. Joel and the team designed a study to determine the perceptions of both parents and students of Laisamis Secondary School (LSS) about formal education, gender roles in leadership, and early marriage among pastoral communities in Laisamis, northern Kenya.

The idea that Denna and his colleagues developed for the engagement intervention came from students during an initial consultation meeting with Denna. The school students were keen to discuss their views on HIV, fertility and family planning among young people. They discussed HIV preventive and treatment strategies, natural and modern methods for contraception. Through the examples the young people gave, they began to discuss the problems and benefits of high fertility. The young people Denna talked about his research, and discussed reproductive health with, wanted to share what they were learning with others at school and to tell the community at large about Denna’s research topic. As a result, the young people working with Denna designed an engagement intervention to tell young people at Mwanza Secondary School about the topic of Denna’s research. The intervention activity was a drama, which was performed in front of the young students and evaluated thereafter.

Imelda also worked within a school setting, with the aim of establishing whether the high school students and the communities they came from were aware of the high burden of maternal deaths in the country, the causes of such deaths, who is at risk of death, and the circumstances surrounding such deaths. The idea was not only to provide information but also to establish what they know about maternal surveillance and response cycle with a view to building that knowledge to feed into safe motherhood initiatives as preventive strategies. In addition, Imelda and her colleagues wanted to understand how to empower students and the wider communities to engage more in Imelda’s project to share information about the maternal health project.

### What the CPE activity was

Joel and colleagues trained six field assistants from the community to assist in sample collection and pathogen transmission experiments by camel keds. At the end of the laboratory studies, they organized three scientific data dissemination workshops in Laisamis and Marsabit to share research findings to the pastoralist communities together with other stakeholders including local leaders, County administrators, veterinary officers, and LSS students. As a part of this, they took a total of 100 students (accompanied by their teachers) for field visits to sample camel blood, screen for blood-borne pathogens, collect camel keds, setting traps for biting flies spreading vector-borne diseases including mosquitoes, sand fly vectors of human leishmaniasis, *Stomoxys* spp., among others, so that the young people in the community gained greater insights into the One Health (OH) research project.

In addition, the team held focus group discussions (FGDs) with 14 groups of students to understand their opinions on socio-cultural factors that limit access to education, class performance, and progression to higher levels of education. We also collected their perceptions on bullying, discrimination, physical harassment, and gender roles in leadership and academic performance. In total, 70 students (43 boys and 27 girls) shared information with the team. Joel and his colleagues also conducted a survey to understand the perceptions of parents on gender equality in formal education, leadership, and their opinions on teenage marriage, and school enrolment of both boys and girls.

Denna held an initial consultative meeting with school students to think of ideas for sharing information about the research project. The students suggested two prototype ideas that included a drama and song as the engagement interventions. The two engagement interventions were tested with a selected sample of 412 students. Feedback was openly collected from the audience on whether the language and the content of the activity was easily understood by the target audience, whether the information in the intervention package was trustworthy or credible, whether the intervention type was desired by the target audience and finally whether the intervention package was age, gender, culturally appropriate. Finally, students proposed drama to be the final engagement intervention for rolling-out and evaluation.

Denna and the school students then co-developed the state of art drama with the help from a performing art consultant. Students organized and performed the drama in front of the specifically chosen students as the sample for evaluation study. During the drama they shared information on key concepts on fertility, social economic issues in region with higher fertility, natural and modern family planning methods and the role of science to human development.

Qualitative and quantitative data were collected before and after the final theatre performance. The audience was made up of male and female students in their second and third years of secondary education. The assumption was to compare groups with similar ages. Through drama performance, we expected to increase awareness to the audience by comparing measured estimates of knowledge before and after theatre performance.

Imelda began the work on her engagement activities by obtaining permission from the headmaster and director of studies in the school to meet students in classes not doing national examinations in their year. Once permission was obtained the students were told about the study. Out of 150 students who were available, 81 volunteered to participate in the pre-test. The students were then asked how they would like to disseminate the information they learned through their work. They agreed to use a combination of music, poems and drama.

Imelda set out to establish what the students understood about maternal deaths, the causes and risk factors, the Three Delays ( Barnes-Josiah *et al.*, 1998) associated with maternal deaths and the application of pillars of safe motherhood, plus maternal death reviews as preventive strategies. In addition, Imelda wanted to understand how students and communities could feed into the then ongoing research project to improve implementation of its key outcomes. As the students learnt about preventable maternal deaths, they discussed what they could do in their communities to support maternal death surveillance and response.

Overall, the school students gained knowledge about key aspects regarding causes of maternal deaths and preventive measures such as pillars of safe motherhood, maternal deaths surveillance and response. In addition, the students appreciated that even adolescents can die from many complications related to pregnancy. Importantly, they were able to disseminate messages from what they had learnt to some members of the communities and their parents, which impressed them greatly.

When Imelda’s engagement project was coming to an end, the COVID-19 pandemic had just begun, so Imelda used the engagement opportunities with the students to share information about the threat COVID-19 posed to the community and pregnant women in particular (including challenges resulting from restrictive measures during lockdown), the impact on the quality of care, and the importance of timely access to health services. Thus, prevention of COVID-19 was included as part of the engagement activities.

### What did we learn from the CPE that we did in THRiVE?

Joel found that two-way public engagement and involvement of livestock farmers in field experiments improved the quality of research and provided the opportunity for mutual engagement for informing, educating, and training from farmer to scientist and scientist to farmer. The findings of this work showed that livestock rearing and teenage marriages were the major socio-cultural factors in Laisamis that limited the access to formal education for boys and girls, respectively. The findings were disseminated to parents, students, teachers, and the School’s Board of Management, and a report providing recommendations to guide policy makers was submitted to the Ministry of Education - Marsabit, for improved school enrollment and for subsequent progression to higher levels of education.

Furthermore, by working with young people in the field research, Joel and his colleagues found the interest and encouragement to become innovative and creative thinkers; one group of students came to Joel with a fly trap they had designed – based on the work they had done in the project.

Denna also found value in talking within the school about his research – and gaining more knowledge on the context of his research from these interactions. He saw value in the engagement before, during and after research, in terms of ensuring community members understand what research is being done, but also in being able to share their ideas on the topic.

Imelda learnt through her engagement in the schools that students and their families had limited information about conditions that kill mothers and became pillars of safe motherhood. Most students at first thought that young people were not at risk of maternal deaths, except if they induced an abortion. Their knowledge of causes of pregnancy-associated deaths improved by the end of the activity and they portrayed themselves as having been greatly empowered since they are the fathers and mothers of tomorrow, they would know what to do in addition to helping other members of the community. This was an important learning for Imelda who had originally thought of the young people as being channels for sharing information with adults rather than being themselves key beneficiaries.

## Recommendations

From this experience, we recommend:

1.  Making engagement with study participants an integral part of all research studies from start to finish. Listening to what people in the community have to say about the study focus can, as Joel found out, refocus study objectives to address community priorities. 

2.  Community and public engagement activities not only allow those who take part in these activities to be informed by the researchers and vice versa, but they can also feel empowered to inform others and make use of research results themselves to influence practice and policy discussions.

3.  Working closely with young people to talk about our research topic allowed Imelda, Denna and Joel to gain insights into the local understandings of science, and the response to official messaging about health.

4.  Community engagement activities cost money – we recommend that this is costed into research projects; indeed, many funders now welcome such items in the budget lines. Communities and the public readily enhance their knowledge about ongoing research, which may empower them to inform others, and they can be quick channels of disseminating research findings to a wider audience.

We also learnt to be patient because sharing knowledge and information takes time, time that we may feel should have been spent on ‘our’ research. We recognise the enthusiasm and interest exhibited by the students as an important outcome of the projects. We hope that this experience will be a foundation for the students’ interest and engagement in science and research as the young people grow into adults. We are all committed to delivering the best science for us and the upcoming researchers.

## Data availability

No data are associated with this article.
