# Case studies from the experience of early career researchers in East Africa in building community engagement in research

**DOI:** 10.12688/aasopenres.13349.2

**Published:** 2022-06-27

**Authors:** Joel L. Bargul, Denna M. Mkwashapi, Imelda Namagembe, Immaculate Nakityo, Annettee Nakimuli, Josaphat Byamugisha, Daniel Semakula, Janet Seeley, Nelson K. Sewankambo

**Affiliations:** 1 Animal Health Theme, International Centre of Insect Physiology and Ecology (icipe), Nairobi, P.O. Box 30772-00100, Kenya; 2 Department of Biochemistry, Jomo Kenyatta University of Agriculture and Technology (JKUAT), Nairobi, P.O. Box 62000-00200, Kenya; 3 THRiVE, c/o School of Medicine, Makerere University College of Health Sciences, Kampala, P.O. Box 7072, Uganda; 4 National Institutes for Medical Research, Mwanza, P.O. Box 1462, Tanzania; 5 School of Medicine, Makerere University College of Health Sciences, Kampala, P.O. Box 7072, Uganda; 6 Mulago Specialized Women and Neonatal Hospital (MSWNH), Kampala, P.O Box 7272, Uganda; 7 Department of Global Health and Development, London School of Hygiene and Tropical Medicine, London, WC1H 9SH, UK

**Keywords:** Public engagement, health research, Kenya, Tanzania, Uganda, East Africa

## Abstract

**Background:** In this paper, we explain how three early career researchers actively engaged community members in their health research projects in Kenya, Tanzania and Uganda, and what was learnt from the experience. The research project in Kenya was on camel trypanosomiasis and the role of camel biting keds (or louse flies) in disease transmission. The project in Tanzania looked at the effect of human immunodeficiency virus and antiretroviral therapy on fertility and ascertained the trends in the use of family planning services amongst women of reproductive age. The focus of the project in Uganda was the implementation of maternal death surveillance and the response policy to determine the cause of maternal deaths and how they might be prevented.

**Methods:** In the three different settings, efforts to ensure local community engagement provided a focus for the researchers to hone their skills in explaining research concepts and working in partnership with community members to co-develop ideas, their research methods and outputs.

**Results: **Involvement of communities in scientific research, which entailed a two-way mutual engagement process, led to (i) generation of new research ideas that shaped the work, (ii) strengthened mutual trust, and (iii) promoted uptake of research findings.

**Conclusion:** Our key findings strongly support the need for considering community engagement as one of the key components in research studies.

## Disclaimer

The views expressed in this article are those of the author(s). Publication in AAS Open Research does not imply endorsement by the AAS.

## Introduction

Over the past decade, there has been a growing awareness of the value of engaging local communities and the wider public in research. The coronavirus disease 2019 (COVID-19) pandemic has highlighted how essential the public understanding of science can be to the acceptance of measures to curtail the spread of infection, with disinformation, often through social media, needing to be countered by authoritative and clearly expressed messages from researchers, epidemiologists, virologists and behavioural scientists throughout the world ( Larson, 2020; Plohl & Musil, 2021; Provenzi & Barello, 2020). For many scientists engaged in the rapidly evolving fields of COVID-19 research, the need to respond to media requests for information or explain their findings to particular interest groups, has become an important role in the effort to counter the spread of infection and encourage vaccine uptake ( Safford *et al.*, 2021; Umviligihozo *et al.*, 2020). The societal value placed on funding, doing and sharing the outputs of scientific research is nurtured through effective community and public engagement ( Holzer *et al.*, 2014).

Many different terms have been used to describe the involvement, the engagement and the participation of people from the community in which research takes place, and the wider public. Involving local community members in research can improve the relevance and quality of research, as those directly affected by the subject under study, for example an infectious disease, can draw attention to factors in the local environment which can enhance the usefulness of the research ( Tindana *et al.*, 2007).

We draw on the definition of public engagement used by Cohen *et al.* (2008: 2): ‘a process that provides people with trustworthy information on key policy issues, elicits their input, and integrates it into decision-making and social action’. They make a distinction between this broader engagement agenda and that of ‘community engagement’, where the people directly participating in or affected by a research project are the focus of engagement. Both are important, and we would argue that as an introduction to broader ‘public engagement’, local community engagement provides a focus for emerging scientists to hone their skills in explaining research concepts and to work with community members to develop ideas, methods and outputs ( MacQueen *et al.,* 2015; Musesengwa *et al.,* 2018; Tembo *et al.,* 2021).

In addition, there is an increasing awareness by research funders of the importance of community and wider public engagement, and for researchers to include a public engagement component in grant applications. The case is made in grant calls for public involvement in research to serve ‘broader democratic principles of citizenship, accountability and transparency’ ( National Institute of Health Research, 2021) and increase public trust ( African Academy of Sciences, 2021). This pressure to include an engagement component in research projects can seem particularly daunting to early career researchers, embarking on an independent research project for the first time. Community engagement has a long history within the space of international development, which provides many examples on which to build. Guidance for researchers exists, in an effort to demystify the terminology and encourage greater involvement in research as well as good practice ( African Academy of Sciences, 2021; Wellcome Trust, 2021; https://www.unicef.org/mena/reports/community-engagement-standards; https://www.nihr.ac.uk/documents/resource-guide-for-community-engagement-andinvolvement-in-global-health-research/27077).

In this paper we explain how three early career researchers actively engaged community members in research in Kenya, Tanzania and Uganda, and what we learnt from the experience. While the studies were affected by the COVID-19 pandemic, the work proceeded during the pandemic, albeit at a slower pace due to travel restrictions, among other guidelines put in place by the governments to control the spread of the disease ( Ministry of Health Uganda, 2020).

Our research was supported through the Training Health Researchers into Vocational Excellence in East Africa ( THRiVE) project which is a collaborative research capacity building project involving five universities (Makerere University - Uganda, Gulu University - Uganda, Kilimanjaro Christian Medical University College - Tanzania, University of Cambridge – United Kingdom, UK, London School of Hygiene and Tropical Medicine - UK); and three research institutes (Uganda Virus Research Institute, International Centre of Insect Physiology and Ecology in Kenya and the National Institute of Medical Research, in Mwanza - Tanzania). The goal of THRiVE was to develop a critical mass of world-class researchers and research leaders capable of conducting high quality independent research and transforming communities where they live and work.

Our approach has been to build a cohort of researchers, providing support and mentoring to scientists at different stages of their research careers, including graduate interns, master’s, doctoral and post-doctoral researchers. We have focused on achieving research excellence in the areas of (a) infectious diseases/neglected tropical diseases, (b) maternal, neonatal and reproductive health, and (c) non-communicable diseases. In total, 70 research fellows have benefitted from supervision, training and mentoring in the THRiVE project across the three East African countries.

We begin by describing the research settings, and the scientific research focus of our three case studies – research led by Joel Bargul, Denna Mkwashapi, and Imelda Namagembe, before recounting the engagement activities undertaken, and our learning from that experience.

### The settings and the background to the research project

***The Joel Bargul study “Camel trypanosomiasis and its transmission in northern Kenya.*”** Joel’s research project in Kenya was on camel trypanosomiasis and the role of camel biting keds (or louse flies, genus *Hippobosca*) in disease transmission. The study was conducted in Laisamis, Marsabit County, about 450 km northeast of Nairobi City. We learnt from the engagement sessions with the livestock farmers and other community members that camels were preferred to other livestock because of their resilience to survive in harsh climates of the arid and semi-arid regions. They are kept for milk, meat, hides, transport, income, and for social capital. However, camel productivity is constrained by ectoparasites, biting flies, and the diseases they transmit. In northern Kenya, little information is available about the diseases circulating in livestock or their possible transmission by keds. Keds also occasionally feed on humans and in the process, they could transmit zoonotic pathogens from the infected animals.

During field-based sampling, Joel engaged the livestock owners and community field assistants in collection of blood and camel keds. Training of the assistants to understand the research project preceded the assignment of sample collection. These assistants were engaged in preliminary screening of freshly collected blood samples for pathogens, and later participated in pathogen transmission experiments by camel keds to determine vector competence. Whenever a diseased sample was identified (i.e. infected with trypanosomes), the camel owner was notified and then allowed to observe the infected sample under the microscope to gain knowledge of the disease. All infected animals from sampled herds were immediately treated at no cost to the farmer. 

Earlier findings from research in this study area showed that various pathogens including *Anaplasma* and *Ehrlichia* spp. and trypanosomes species *Trypanosoma vivax* and *T. evansi* were present in camels and in keds collected from them ( Kidambasi *et al.*, 2020). Therefore, Joel and his team studied the ability of camel keds to transmit trypanosomes and other blood-borne pathogens from naturally infected camels to experimental mice and rabbits via blood-feeding bites by following an established protocol ( Oyieke & Reid, 2003). They demonstrated, for the first time, that camel keds, *Hippobosca camelina,* could transmit ‘ *Candidatus* Anaplasma camelii’ from camels to mice and rabbits, but not trypanosomes ( Bargul *et al.*, 2021).

***The Denna Mkwashapi study “Influence of human immunodeficiency virus (HIV) and antiretroviral therapy (ART) on fertility and uptake of family planning services.*”** The project that Denna undertook for his doctoral studies aimed to understand the effect of HIV and ART on fertility and ascertain the trends in the use of family planning services amongst women of reproductive age in Tanzania.

With the changing of the HIV epidemic to a chronic condition through increased access to ART in Tanzania, it is not clear how HIV has impacted on the fertility gap between HIV infected and uninfected women at different time periods of the ART coverage. Denna and his team wanted to know to what extent uptake of family planning services has been changing with time. For the study, Denna used data from the 25 year old - Magu Health and Demographic Surveillance System (HDSS) ( Kishamawe *et al.*, 2015). The study area lies 20 km east of Mwanza City, the region's capital, with a predominantly rural population. Magu HDSS is comprised of nine villages with a total combined population of 45,000 people in 2020.

The fertility data Denna used were drawn from 35 rounds of household visits from 1994 to 2018, which captured all births in the resident population. HIV status data were drawn from eight rounds of HIV epidemiologic and serologic surveillance, which was conducted every three years, from 1994 to 2018 among all eligible, resident adults aged 15 years and above. Using those data, total fertility rate, age specific fertility rate, general fertility rate, contraceptive use and unmet need for contraception by HIV status and different levels of ART availability were compared over time and factors associated with the changes investigated.

***The Imelda Namagembe study “Implementation of maternal death surveillance and response policy: the impact of training and community engagement.*”** Imelda’s research project in Uganda was based in the Department of Obstetrics and Gynaecology at Mulago National Referral and Teaching Hospital for the Makerere University initially located just north of central Kampala (the capital city of Uganda), but recently moved to Kawempe on the outskirts of northern Kampala. The hospital has one of the busiest labour wards in Africa with 39,000 deliveries a year ( Hughes *et al.*, 2020). The labour ward has about 46 obstetricians and gynaecologists ( Namwaya *et al.*, 2020). Health workers provide Reproductive Maternal Newborn, Child and Adolescent Health Services (RMNCAH) to all women attending the facility from all over Uganda. The National Referral Hospital is faced with a high burden of maternal and perinatal deaths and contributes the highest number of deaths to Kampala district within Uganda ( Ministry of Health, 2019). This is partly due to the hospital being a referral site and most of the deaths are due to conditions that are preventable, such as excessive bleeding, hypertensive disorders of pregnancy (pre-eclampsia/eclampsia), sepsis (bacterial infection) from obstructed labour and abortion-related complications. Serious conditions are compounded by delay in seeking care, delays in getting transport and delays waiting at health facilities ( Kiondo *et al.*, 2021; Nakimuli *et al.*, 2016). Most of the women who die, die young, with a mean age of about 26 years, and of these 10 to 15% are adolescents/young adults (< 20 years) ( Ministry of Health, 2018). One of the strategies to improve maternal and new-born outcomes is to conduct timely Maternal Death Surveillance and Response (MDSR) policy where the hospital staff are expected to notify a maternal death within 24 hours, conduct a death audit to identify gaps in care that contributed to the death within 7 days, develop recommendations and follow up implementation to prevent future deaths. However, this was not being done on a regular basis.

The aim of the study conducted by Imelda as part of THRiVE-funded research was to examine the implementation of MDSR policy for a 3-year period (2016 – 2018) as a baseline to determine the cause of maternal deaths and prevention. This was followed by an exploration of the barriers and facilitators to MDSR implementation and to later evaluate the impact of training with stakeholder engagement on MDSR performance.

In the course of her research, Imelda engaged with both the internal stakeholders at the hospital (health workers and administrators) and external stakeholders (lawyers, representatives from the Ministry of Health, study partners with expertise in reproductive health). The engagement aimed at identifying the barriers to the quality improvement process of MDSR through an exploration of stakeholders’ perspectives and what could be done to improve on the outcomes for mothers and their babies.

All three research projects described above were conducted in community settings – albeit in three very different places: arid northern Kenya, rural north western Tanzania and the main referral hospital in the capital of Uganda. In the next section, we explain where our idea for the community engagement activities in the projects came from.

## Ethical approval

Joel’s study was undertaken in strict adherence to experimental guidelines and procedures approved by the *icipe* Institutional Animal Care and Use Committee, IACUC (REF: IACUC/ICIPE/003/2018). In addition, the study received ethical consent from the Pwani University Ethics Review Committee (REF: ERC/EXT/002/2020) to collect the information for various research activities with livestock farmers – including community engagement. Animals were handled carefully to minimize pain and discomfort during sampling. Permission to engage with pastoralist farmers in research and to sample their livestock was obtained through verbal consent, as most herders were unable to read or write. Engagement of secondary school students in research was conducted after obtaining the permission from Laisamis Secondary School (LSS) principal (the term used for the headmaster/mistress in Kenyan schools). Oral assent was sought from the LSS student volunteers (aged between 15 – 20 years, mean age = 17.32 years) who were provided with sufficient information about the focus group discussions (FGDs) to allow each individual to make informed and independent decisions to participate in the survey. Engagement through FGDs posed minimal risk to the students, thus permission was not sought from their parents, but from the school principal, who granted approval to our request and then linked us to the students. The main ethical challenge that Joel faced in his study was the lack of clear strategies on how to give credit to the many community members who contributed to new study ideas during community engagement meetings.

Denna’s project targeting women of reproductive age in Tanzania to determine the influence of HIV and ART on fertility and the uptake of family planning services received ethical approval from the Review Committee of Kilimanjaro Christian Medical College of the Tumaini University of Tanzania (certificate number 2440). This approval covers community engagement activities. Permission to work with secondary school students was granted by the director, Mwanza City Council (REF: MCC/SE/20.VOL.II/127). The secondary school students, who were aged 18 years and above, provided verbal consent to participate in the consultative meetings, drama, and debate activities. 

Imelda’s study received approval from the Makerere University School of Medicine Higher Degrees Research and Ethics Committee (SOMREC REF: 2018-001) and Uganda National Council for Science and Technology (UNCST REF: SS4797) to conduct the research on MDSR and community engagement. Engagement of secondary school students in research was preceded by permission from the headmaster and the director of studies. In addition, approval to engage with students in Uganda was obtained from the Ministry of Education and Sports (Uganda). Further, there was a waiver of consent from parents since the research was minimal risk. The students who participated in drama activities and qualitative interviews provided written consent for those aged 18 years and above, whereas assent was obtained for those aged below 18 years. The age range of the secondary school students was 16 – 21 years, with a mean age of 17.7 years. The students were keen to have their project completed before their final exams, which had been shifted to April 2021 (because of the COVID-19 pandemic).

### Where did the idea for the community engagement focus come from?

Joel’s work was firmly embedded in the day-to-day processes of camel herding, so the goodwill of the herders was critical to the success of the project. Joel had ensured that the key stakeholders, particularly the camel farmers, were familiar with the focus of the research and encouraged the exchange of ideas. The farmers used to freely discuss the key challenges they faced during livestock production, ranging from animal husbandry practices, the burden of pests and diseases, the transmission of diseases, and the traditional and modern ways used for control. Joel knew the work that he and his colleagues conducted would – if successful – help to address these pest and disease challenges.

However, it was another area of daily life that Joel chose to focus his community engagement activities. During field visits to collect samples, the team commonly observed that children of school-going age were not enrolled in school, but engaged in other duties at home, such as livestock herding activities. Joel wanted to understand more about the value placed on education for children and the contribution of children to labour (including camel herding) with a view to trying to support great opportunities for the children to go to school. Joel and the team designed a survey to determine the perceptions of both parents and LSS students towards formal education, gender roles in leadership, and early marriages among pastoral communities in Laisamis, northern Kenya.

The following two video links summarize the engagement activities that Joel undertook in Laisamis, Marsabit County, for his research studies on camel health ( https://www.youtube.com/watch?v=FuM-RUnjpwM), and access to education by the children from the nomadic pastoralist communities ( https://vimeo.com/531249510).

The idea that Denna and his colleagues developed for the engagement intervention came from students during an initial consultation meeting with Denna. The school students were keen to discuss their views on HIV, fertility and family planning among young people. They discussed HIV preventive and treatment strategies, and natural and modern methods of contraception. Through the examples the young people gave, they began to discuss the problems and benefits of high fertility. The young people Denna talked about his research, and discussed reproductive health with, wanted to share what they were learning with others at school and to tell the local community at large about Denna’s research topic. As a result, the young people working with Denna designed an engagement intervention to tell young people at Mwanza Secondary School about the topic of Denna’s research. The intervention activity was a drama sketch, which was performed in front of the young students and evaluated thereafter.

Imelda also worked within a school setting, with the aim of establishing whether the high school students and the communities they came from were aware of the high burden of maternal deaths in the country, the causes of such deaths, who is at risk of death, and the circumstances surrounding such deaths. The idea was not only to provide information but also to establish what they know about maternal surveillance and response cycle with a view to building that knowledge to feed into safe motherhood initiatives as preventive strategies. In addition, Imelda and her colleagues wanted to understand how to empower students and the wider communities to engage more in Imelda’s project to share information about maternal health project.

### What the community engagement activity was

Joel and his team have been conducting community engagement activities with livestock farmers and members of the local community during field visits for sample collection. In addition, as noted above, he engaged LSS students in research and mentorship programmes.

At the end of the laboratory studies, Joel and his team organized three scientific data dissemination workshops in Laisamis and Marsabit, northern Kenya, to share research findings with the pastoralist communities and other stakeholders, namely local leaders, County administrators, veterinary officers, and LSS students. As a part of a training workshop, they took a total of 100 students (accompanied by their teachers) for field visits to expose them to sample collection strategies and preliminary analysis. The following samples were collected: camel blood for onsite screening of selected blood-borne pathogens using microscopy, camel keds, ticks, mosquitoes, sand flies, and stable flies. Students were trained on how to prepare preserved insect collections using the field-collected samples for long-term use, for instance in biology practical lessons and as teaching materials for classroom demonstrations. Oral presentations combined with practical sessions were organized to train students about the various insect species they collected from the field and the roles the insects played in transmission of vector-borne diseases. This engagement and practical exposure to scientific research stimulated curiosity in the young learners as evidenced by their active participation during training and the many questions they raised, and could motivate them to perform better in class. Regular engagements of young students in advanced scientific research exposes them to real life applications of science among other taught subjects, thus in the longer term promoting science, technology and innovation.

In another engagement study with students of LSS, Joel and his team conducted FGDs with 14 groups of students to understand their opinions on socio-cultural factors that limit access to education, class performance, and progression to higher levels of education. The perceptions of students on bullying, discrimination, and physical harassment at school, and also the gender roles in leadership and academic performance were discussed. In total, 70 students (43 boys and 27 girls) shared information with the team. The student participants were categorized based on sex and the year of study from 1 ^st^ to 4 ^th^ year (of secondary education).

Joel and his colleagues also conducted a survey to understand the perceptions of parents (n=384 households) on gender equality in formal education, leadership, and their opinions on teenage marriage, and school enrolment of both boys and girls. Dissemination meetings were conducted in Laisamis, Marsabit County, in November 2020 to inform key education stakeholders about the objectives of the study and how it would benefit the pastoral communities.

Denna held an initial consultative meeting with school students to think of ideas for sharing information about the research project. The co-designed ideas that the students preferred most included a drama sketch and song as engagement interventions. The two engagement interventions were tested with a selected sample of 412 students. Feedback was openly collected from the audience on whether the language and the content of the activity was easily understood by the target audience, whether the information in the intervention package was trustworthy or credible, whether the intervention type was desired by the target audience and finally whether the intervention package was age, gender, culturally appropriate. Finally, students proposed that a drama sketch to be the final engagement intervention for rolling-out and evaluation.

Denna and the school students then co-developed the state of art drama with the help from a performing art consultant. Students organized and performed the drama in front of the specifically chosen students as the sample for the evaluation study. During the drama they shared information on key concepts on fertility, social economic issues in region with higher fertility, natural and modern family planning methods and the role of science to human development.

Qualitative and quantitative data were collected before and after the final theatre performance. The audience was made up of male and female students in their second and third years of secondary education. The assumption was to compare groups with similar ages. Through the drama performance, we expected to increase awareness to the audience by comparing measured estimates of knowledge before and after the performance.

Imelda began the work on her engagement activities by obtaining permission from the headmaster and director of studies in the school to meet students in classes not doing national examinations in their school year. Once permission was obtained the students were told about the study. Out of 150 students who were available, 81 volunteered to participate in the pre-test. The students were then asked how they would like to disseminate the information they learned through their work. They agreed to use a combination of music, poems and drama.

Imelda set out to establish what the students understood about maternal deaths, the causes and risk factors, the Three Delays ( Barnes-Josiah *et al.*, 1998) associated with maternal deaths and the application of pillars of safe motherhood, plus maternal death reviews as preventive strategies. In addition, Imelda wanted to understand how students and communities could feed into the then ongoing research project to improve implementation of its key outcomes. As the students learnt about preventable maternal deaths, they discussed what they could do in their communities to support maternal death surveillance and an appropriate response.

Overall, the school students gained knowledge about key aspects regarding causes of maternal deaths and preventive measures such as pillars of safe motherhood, and MDSR. In addition, the students appreciated that even adolescents can die from many complications related to pregnancy. Importantly, they were able to disseminate messages from what they had learnt to some members of the communities and their parents, which impressed their families greatly.

When Imelda’s engagement project was coming to an end, the COVID-19 pandemic had just begun. So Imelda used the engagement opportunities with the students to share information about the threat COVID-19 posed to the community and pregnant women in particular (including challenges resulting from restrictive measures during lockdown), the impact on the quality of care, and the importance of timely access to health services. Thus, prevention of COVID-19 was included as part of the engagement activities.

Imelda obtained permission to access the secondary school students of St. Aloysius Senior Secondary School (mixed school with day and boarding students). Then, Imelda and Immaculate (coordinator) from THRiVE had discussions with 150 students to inform them about the school engagement plan focusing on maternal health. The lead role of the students was emphasized since it was conceived as student-centred project. Through a brainstorming session, they agreed on the methods used to co-create/ co-design their prototypes (i.e the drama-skit, song and poem). At that same meeting, 6-student champions (3 males and 3 females) were nominated and voted for to work closely with the head prefects to mobilize the fellow students. It was at that point when students who felt comfortable to do the pre-test were requested to register voluntarily. The pre-test was offered at a follow up visit (two weeks later) after which Imelda made a simplified presentation about her research. The pre-test was composed of semi-structured questions on: the definition of research, importance of maternal health research; definition of a maternal death; burden of maternal deaths (national maternal mortality ratio, number of maternal deaths country wide in simple terms), causes of maternal deaths, who is at risk of death, and prevalence of teenage pregnancy. They were asked a few questions about what should be done if a health worker loses a pregnant woman when on duty; steps for maternal death surveillance and pillars of safe motherhood as strategies to prevent maternal and newborn deaths. There was a question-and-answer session after the pre-test to enhance the understanding of various aspects.

After the pre-engagement test, the students received pamphlets containing information on the various topics that were assessed in the test. They were encouraged to read more, ask questions (asking teachers, searching the internet, and consulting members of the community where possible). The plans for the follow-up activities were discussed, the students worked closely with four teachers (two ladies and two men). A music/drama expert joined the team to help fine-tune their ideas. They continued with practise in the evenings. At the climax of events, around 18th-March 2020, lock down due to COVID-19 pandemic was suddenly put in place. Schools closed for 6-months, and re-opened in October 2020 for students sitting national examinations. Engagement activities resumed in December 2020 after end of term examinations. By then we had only 100 students in the age range 16 to 21 years. The students really wanted to complete the maternal health project, so they proposed dedicating 10 days to practise. The period of upkeep was facilitated by funds from a grant after discussions with the principal of St. Aloysius Senior Secondary School and the Director THRiVE. We were given permission to present to the communities after a mass/service respecting COVID-19 guidelines ( Ministry of Health Uganda, 2020). At that time, only 70 people were allowed in the parish church. We were situated in a big compound next to the church, using two 100-seater tents (the students were in one tent and other members of the community in another), masks were given to all, sanitizers put in place and a distance of a minimum of 2 metres between people ensured. We had an average of 40 adult participants per session. Each performance lasted about 20 minutes, with intervals of 40 – 50 minutes as we waited for another group of people to come from church. Three performances were done on that Sunday, to a total of 120 people. We received pre-packed snacks and sodas. Another showcase for the maternal project was conducted on the students’ request, when their parents / guardians were allowed to be at school for blessing prayers before examinations in March 2021. Again COVID-19 prevention guidelines were respected. None of the students suffered COVID-19 related illnesses. 

### What did we learn from the community engagement that we did in THRiVE?

Joel found that the two-way community engagement and involvement of livestock farmers in field experiments improved the quality of research and provided the opportunity for mutual engagement for informing, educating, and training from farmer to scientist and scientist to farmer. The findings of this work showed that livestock rearing and teenage marriages were the major socio-cultural factors in Laisamis that limited the access to formal education for boys and girls, respectively. The findings were disseminated to parents, students, teachers, and the School’s Board of Management, and a report providing recommendations to guide policy makers was submitted to the Ministry of Education - Marsabit, focusing on how to improve school enrollment and encourage progression to higher levels of education.

Furthermore, by working with young people in the field research, Joel and his colleagues found that the students were encouraged to become innovative and creative thinkers: one group of students came to Joel with a fly trap they had designed – based on the work they had done in the project.

Denna also found value in talking within the school he worked with about his research – and gaining more knowledge on the context of his research from these interactions. He saw value in the engagement before, during and after research, in terms of ensuring community members understand what research is being done, but also had an opportunity to be able to share their ideas on the topic.

Imelda learnt through her engagement in the schools that students and their families had limited information about conditions that kill mothers and the research engagement activities empowered them with knowledge that they promised to share by becoming champions for safe motherhood. Most students at first thought that young people were not at risk of maternal deaths, except if they induced an abortion. Their knowledge of causes of pregnancy-associated deaths improved by the end of the activity and they portrayed themselves as having been greatly empowered since they are the fathers and mothers of tomorrow. They felt empowered on issues of preventing teenage pregnancy, other maternal health issues. Some shared testimonies of having advised their pregnant sisters and friends on early seeking of antenatal services and health care when the labour started at the time when lockdown measures for COVID-19 control were in place. This was an important learning for Imelda who had originally thought of the young people as being channels for sharing information with adults rather than being themselves key beneficiaries. Together, our findings from the community engagement provide key information that could inform policy.

## A limitation of the study

Our case studies would have benefitted from a more rigorous approach to measuring and evaluating the socio-economic impact of the community engagement activities. However, monitoring and evaluation of the engagement outcomes were not conducted due to the limited funding available.

## Recommendations

From this experience, we recommend:

1. Making engagement with study participants an integral part of all research studies from start to finish. Listening to what people in the community have to say about the study focus can, as Joel found out, refocus study objectives to address community priorities.

2. Community engagement activities not only allow those who take part in these activities to be informed by the researchers and vice versa, but they can also feel empowered to inform others and make use of research results themselves to influence practice and policy discussions, leading to wider public engagement.

3. Working closely with young people to talk about our research topic allowed Imelda, Denna, and Joel to gain insights into the local understandings of science, and the response to official messaging about health.

4. Community engagement activities cost money – we recommend that this is costed into research projects; indeed, many funders now welcome such items in the budget lines. Communities and the wider public with increased knowledge about ongoing research may be empowered to inform others., Community members who share information gained through their engagement in research can aid reaching out to the general public for example in disseminating research findings to a wider audience, especially to those under resource-challenged settings. Other potential opportunities for efficient scaling up of engagement activities, for instance during pandemics (i.e. COVID-19) to include using social media platforms such as Facebook, WhatsApp, and Twitter as most community members in these studies have access to mobile phones, and also using the mass media (e.g. newspaper articles, opinion pieces, discussions on radio and television).

We also learnt to be patient because sharing knowledge and information takes time, time that we may feel should have been spent on ‘our’ research. We recognise the enthusiasm and interest exhibited by the students as an important outcome of the projects. We hope that this experience will be a foundation for the students’ interest and engagement in science and research as the young people grow into adults. We are all committed to delivering the best science for us and the researchers of the future.

## Data availability

No data are associated with this article.
