# Supplementary material for: Case studies from the experience of early career researchers in East Africa in building community engagement in research
Source: AAS Open Res. 2022 Jun 27;5:13. Originally published 2022 Mar 16. [Version 2] doi: 10.12688/aasopenres.13349.2 (PMC10541536; doi:10.12688/aasopenres.13349.2)
Supplement: Peer review history

# Reviewer report: Reviewer response for version 2

**DOI:** 10.21956/aasopenres.14581.r30085

**Published:** 2023-09-29

**Authors:** Nadia Tagoe

**Affiliations:** 1 Kwame Nkrumah University of Science and Technology (KNUST) in Ghana, Kumasi,, Ghana

The article presents the community engagement experiences of three health researchers undertaking studies in different settings in Africa. The paper seeks to highlight as aspect of health research which is usually not the primary focus of researchers although critical to the health process. The authors indicate how the community engagement activities were conceptualised and implemented and shared the lessons learnt during the process. The strengths of the paper include sharing learning across contextual heterogeneity in terms of settings, study focus and study participants, and clearly outlining actionable recommendations for readers.

 I have a few comments for the authors to consider.

**Introduction:**

- A clear definition of community engagement should be included as the information only indicates how it is distinct from public engagement and not what it is.
- After stating the distinction between community and public engagement, the authors should clearly state which type of engagement their work focused on.

**Ethical approval:**

- Joel’s engagement on formal education, gender and leadership done through a survey and FGDs is a study on its own. Was ethical approval obtained for it?

**Idea for the community engagement focus:**

- The authors need to clearly and consistently state the objective of the community engagement activity, the methods used (i.e., how they engaged the community), the results and lessons learned for all the cases studies. For example, Page 8 states that “the findings were disseminated to parents, students, teachers, and the School’s Board of Management”. How was that done?
- The authors need to differentiate between conducting a survey and FGDs (which is data collection) and community engagement; and the specific characteristic of the activities that make them community engagement (e.g. two-way). For instance, Denna’s community engagement activity which was co-designed by the participants (students) during a consultative meeting is a clear example of community engagement.

**Benefits and challenges of community engagement:**

- I suggest that the authors highlight the benefits and challenges of the community engagement activities (to both the community and researchers) in the paper. Several examples of benefits are demonstrated including health education, and training on specific areas, skills building, identification of other community priorities, promoting science, technology and innovation, and community/school projects such as developing drama activities.  

**Reference differing views and opinions:**

- I suggest outlining the limitations and disadvantages of the three approaches to community engagement and the activities undertaken.

Does the article adequately reference differing views and opinions?

Partly

Are all factual statements correct, and are statements and arguments made adequately supported by citations?

Yes

Is the Open Letter written in accessible language?

Yes

Where applicable, are recommendations and next steps explained clearly for others to follow?

Yes

Is the rationale for the Open Letter provided in sufficient detail?

Yes

Reviewer Expertise:

Health research systems, capacity strengthening, equity and inclusion in research

I confirm that I have read this submission and believe that I have an appropriate level of expertise to confirm that it is of an acceptable scientific standard.

# Reviewer report: Reviewer response for version 2

**DOI:** 10.21956/openresafrica.14581.r29256

**Published:** 2022-07-11

**Authors:** Doreen Tembo

**Affiliations:** 1 Wessex Institute, Faculty of Medicine, University of Southampton, Southampton, UK

I have now had a chance to review the revisions and can confirm that with the exception of a minor revision below, I am happy with the responses.

 Minor typo:

 Please note that the National Institute ***for*** Health ***and Care*** Research is still wrongly referred to as the National Institute ***for*** Health Research.

Does the article adequately reference differing views and opinions?

Partly

Are all factual statements correct, and are statements and arguments made adequately supported by citations?

Partly

Is the Open Letter written in accessible language?

Partly

Where applicable, are recommendations and next steps explained clearly for others to follow?

Yes

Is the rationale for the Open Letter provided in sufficient detail?

Yes

Reviewer Expertise:

Mixed-methods Research, Community Engagement and Involvement Specialist, Health Policy, Health Systems

I confirm that I have read this submission and believe that I have an appropriate level of expertise to confirm that it is of an acceptable scientific standard.

# Reviewer report: Reviewer response for version 1

**DOI:** 10.21956/aasopenres.14494.r29176

**Published:** 2022-04-27

**Authors:** Inbarani Naidoo

**Affiliations:** 1 Human and Social Capabilities Division, Human Sciences Research Council, Pretoria, South Africa

This is a case study approach implemented in three different African settings in Kenya, Tanzania and Uganda and covered vector born disease, HIV and cause of maternal death. That authors differentiate community engagement from public engagement. Community engagements were undertaken with school going children from schools in research catchment area. The engagements are described from the perspective of three early career researchers.

 Below are some reflections and suggestions:

**Title:**

- I recommend reference to the case study approach in the title.

**Use of capital letters mid-sentence:**

- Introduction, page 3: “Masters” is capitalized.
- Page 5, paragraph 2: Total, Age, General.

**Use of abbreviations at first mention:**

- MERS, antiretroviral therapy, Laisamis Secondary School.

**Use of abbreviations at only mention:**

- Total fertility rate, age specific fertility rate, general fertility rate, One Health are mentioned once hence no need for abbreviations to be used for these.

**Consistency:**

- Consistency in referring to authors in text: first names used at times, then both first and last names are used at other times in text.
- Consistency in referring to high school students and secondary school students.
- Consistency in referring to school principal and headmaster.

**Grammar:**

- I suggest a grammar check. 

e.g. "Therefore, the aim of this study Joel led as a part of THRiVE postdoctoral research fellowship was to determine disease transmission patterns among co-herded livestock and study the role of keds in their spread."

**Suggest rewording:**

- Page 4: Camels are preferred to other livestock in many communities because of their resilience to survive in harsh climates with prolonged droughts **downstream of global warming**.
- The authors write that the “study was conducted in Laisamis, Marsabit County, about 450 km northeast of Nairobi City” and then add “field sampling was done in Laisamis located in the south of Marsabit County.”
- "… **laboratory animals** were transported to the field sampling sites in northern Kenya to provide bloodmeals for freshly collected camels keds and thereafter the samples from **those laboratory mice and rabbits** were used to identify transmitted ked-borne pathogens using molecular assays at icipe (Nairobi)."

**Clarity:**

- I suggest explaining what is meant by "co-herded".
- Page 5: health workers are mentioned separately in the surveillance study. Are the health workers different from doctors, midwives, nurses?

**Repetition:**

- The authors list the ethical approvals on page 3 and repeat on page 4 that “ethical permissions were already in place”.

**References:**

- Reference to results published elsewhere should be stated clearly in text Page 4: does the below mean the results of the study are reported in Kidambasi *et al.*, 2020?

"The findings of **this study** show that *Anaplasma* and *Ehrlichia* spp. and trypanosomes species *Trypanosoma vivax* and *T. evansi* are present in camels and in keds collected from them, suggesting a possible role in disease transmission (Kidambasi et al., 2020)."

**Ethical approval for community engagement with school children. Informed consent for community engagement with school children, parents/care givers and/or school management:  **

- The authors state that permission from school principals was obtained. Does the research ethics approvals obtained for all three case studies cover the community engagements? This should be clarified.
- Please state the ethical processes followed to gain access to school children in the community engagements i.e. was consent/assent sought directly from the school children prior to their participation? How were parents engaged to gain access to the school children?

**Methods described to carry out the engagements for the case studies:**

- The section on the settings and background to the research projects can be shortened to emphasize important aspects related to the public/community engagements. For example in the Kenya study the authors state a cross-sectional study design and convenient sampling of camel keds. How does this impact on the case studies for community engagement? What dates were these samples collected?
- What were the ages of the school children in the community engagements?
- A clear distinction should be made for all three case studies as to which aspects were public engagement and which were community engagement.
- The authors state they "designed a study” to assess perceptions of parents and students in Kenya (page 5) and then “developed” an "engagement intervention” referring to an “intervention activity” on page 6. The authors also “worked within a school setting” to assess awareness of maternal deaths on page 6.
- Sufficient detail should be given on the processes and methods for community and/or public engagement in the three country settings to be able to replicate such case studies.

**Reference differing views and opinions:**

- I suggest expanding on ethics in community and public engagement.
- I suggest linking the co-development of ideas, research methods and outputs to a theoretical framework such as the co-design approach alluded to in the abstract.

**Lessons learnt and recommendations:**

- The authors differentiate community engagement from public engagement in the introduction. The authors refer to two-way *public engagement* on page 7 and community engagement in other sections. Aspects of community and public engagement should be clearly described and/or differentiated if applicable, in the three case studies.
- Are *study participants* mentioned in the recommendations on page 7, part of the community or public engagements?
- I suggest highlighting the ethical considerations for such engagements.
- How can the community engagement be scaled up to broader public engagement in resource-challenged and pandemic settings?

Does the article adequately reference differing views and opinions?

No

Are all factual statements correct, and are statements and arguments made adequately supported by citations?

Partly

Is the Open Letter written in accessible language?

Partly

Where applicable, are recommendations and next steps explained clearly for others to follow?

No

Is the rationale for the Open Letter provided in sufficient detail?

Partly

Reviewer Expertise:

Epidemiology, public health research

I confirm that I have read this submission and believe that I have an appropriate level of expertise to confirm that it is of an acceptable scientific standard, however I have significant reservations, as outlined above.

# Response

**Published:** 2022-06-21

**Authors:** Joel L. Bargul

This is a case study approach implemented in three different African settings in Kenya, Tanzania and Uganda and covered vector borne disease, HIV and cause of maternal death. That authors differentiate community engagement from public engagement. Community engagements were undertaken with school going children from schools in research catchment area. The engagements are described from the perspective of three early career researchers. Below are some reflections and suggestions:

**Title:**

- I recommend reference to the case study approach in the title.

We agree and have rephrased the title to read: *“Case studies from the experience of early career researchers in East Africa in building community engagement in research”*

**Use of capital letters mid-sentence:**

- Introduction, page 3: “Masters” is capitalized.

The word *Masters* is now correctly written as: *master’s* (It begins with lower case letter, and has apostrophe). Page 4 under the *“Introduction”* section of revised article.

- Page 5, paragraph 2: Total, Age, General.

We have now written these three words correctly by beginning with lower case letters, i.e. *total, age, general. *Page 5 under the subheading *“The Denna Mkwashapi study”* of revised article.

**Use of abbreviations at first mention:**

- MERS, antiretroviral therapy, Laisamis Secondary School.

In the process of rewriting the background information of the study to refocus it on community engagement as recommended by the reviewer, we deleted the sentence containing the abbreviation ‘MERS’ (Middle East respiratory syndrome coronavirus). We have given the abbreviation of ‘antiretroviral therapy’ as ART at first use in the main text on page 4 (under the subheading *“The Denna Mkwashapi study”*), and have just used ART on page 4. We have also included the abbreviation at first usage of ‘Laisamis Secondary School (LSS)’ on page 5.

**Use of abbreviations at only mention:**

- Total fertility rate, age specific fertility rate, general fertility rate, One Health are mentioned once hence no need for abbreviations to be used for these.

We have deleted the abbreviations TFR, ASFR, GFR, and OH

**Consistency:**

- Consistency in referring to authors in text: first names used at times, then both first and last names are used at other times in text.

To maintain consistency, the names of the researchers are first written in full, first and surname (page 4; last paragraph of “Introduction” of the revised paper), and thereafter they are referred consistently by the first name. Also, we have named each case study by the researcher’s name, i.e. *“The Denna Mkwashapi study “Influence of human immunodeficiency virus (HIV) and antiretroviral therapy (ART) on fertility and uptake of family planning services.”* (see page 4 – 5).

- Consistency in referring to high school students and secondary school students.

We have opted to use ‘secondary school students’ consistently throughout the paper.

- Consistency in referring to school principal and headmaster.

In Kenya, “principal” is commonly used to mean the teacher in charge of a school, unlike in Uganda that prefers “headmaster/mistress” or “head teacher” We have clarified this in the revised paper by stating at first mention: *“…principal* *(the term used for the headmaster/mistress in Kenyan schools)* (Page 5, 1 ^st^ paragraph under “ *Ethical approval”* section).

**Grammar:**

- I suggest a grammar check, e.g. "Therefore, the aim of this study Joel led as a part of THRiVE postdoctoral research fellowship was to determine disease transmission patterns among co-herded livestock and study the role of keds in their spread."

We have refocused the background information to this case study to emphasize community engagement as rightly suggested by the reviewer (page 4 – 5, under *“The settings and the background to the research project”*). Therefore, the above statement has been deleted in the revised article.

**Suggest rewording:**

- Page 4: Camels are preferred to other livestock in many communities because of their resilience to survive in harsh climates with prolonged droughts **downstream of global warming**.

We deleted this sentence in the process of rewriting the background information.

- The authors write that the “study was conducted in Laisamis, Marsabit County, about 450km northeast of Nairobi City” and then add “field sampling was done in Laisamis located in the south of Marsabit County.”

We have deleted this sentence and rewritten the background information.

- "… **laboratory animals** were transported to the field sampling sites in northern Kenya to provide bloodmeals for freshly collected camels keds and thereafter the samples from **those laboratory mice and rabbits** were used to identify transmitted ked-borne pathogens using molecular assays at icipe (Nairobi)."

We have deleted this sentence to emphasize community engagement as suggested by the reviewer.

**Clarity:**

- I suggest explaining what is meant by "co-herded".

We have deleted the sentence containing the word ‘co-herded’

- Page 5: health workers are mentioned separately in the surveillance study. Are the health workers different from doctors, midwives, nurses?

Indeed, health workers are not different from doctors, nurses, and midwives. We have now rephrased this sentence for clarity as follows: *“In the course of her research, Imelda engaged with both the internal stakeholders at the hospital (health workers and administrators) and external stakeholders (lawyers, representatives from the Ministry of Health, study partners with expertise in reproductive health). The engagement aimed at identifying the barriers to the quality improvement process of MDSR through an exploration of stakeholders’ perspectives and what could be done to improve on the outcomes for mothers and their babies.” *(Page 5, 3 ^rd^ paragraph under *“The Imelda Namagembe study…”*)

**Repetition:**

- The authors list the ethical approvals on page 3 and repeat on page 4 that “ethical permissions were already in place”.

To avoid repetition and also refocused this section, we have rewritten the background information. We have therefore deleted the sentence that contained the following clause: *“…ethical permissions were already in place.”*

**References:**

- Reference to results published elsewhere should be stated clearly in text Page 4: does the below mean the results of the study are reported in Kidambasi et al., 2020?

"The findings of this study show that *Anaplasma* and *Ehrlichia* spp. and trypanosomes species *Trypanosoma vivax* and *T. evansi* are present in camels and in keds collected from them, suggesting a possible role in disease transmission (Kidambasi et al., 2020)." Yes, we earlier published the results of this study in Kidambasi et al., 2020 in *AAS Open Research* journal. Full reference: Kidambasi KO, Masiga DK, Villinger J, et al.: Detection of blood pathogens in camels and their associated ectoparasitic camel biting keds, *Hippobosca camelina*: the potential application of keds in xenodiagnosis of camel haemopathogens [version 2; peer review: 2 approved]. *AAS Open Res.* 2020;2:164. 32510036 10.12688/aasopenres.13021.2 7243205 

**Ethical approval for community engagement with school children. Informed consent for community engagement with school children, parents/care givers and/or school management:**

- The authors state that permission from school principals was obtained. Does the research ethics approvals obtained for all three case studies cover the community engagements? This should be clarified.
- Please state the ethical processes followed to gain access to school children in the community engagements i.e. was consent/assent sought directly from the school children prior to their participation? How were parents engaged to gain access to the school children?

**Joel’s** study: Yes, approval by the Pwani University Ethics Review Committee (REF: ERC/EXT/002/2020) provided consent to collect the information for various research activities with livestock farmers - including community engagement. Oral assent was sought from the LSS student volunteers (aged between 15 – 20 years, mean age = 17.32 years) who were provided with sufficient information about the focus group discussions (FGDs) to allow each individual to make informed and independent decisions to participate in the survey. Engagement through FGDs posed minimal risk to the students, thus permission was not sought from their parents, but the school principal who granted approval to our request and then linked us to the students. Page 5, 1 ^st^ paragraph under “ *Ethical approval”* section.

**Denna’s** study: The study, which received ethical clearance, had a component of community engagement. However, research engagement of school children in Tanzania required separate approval from the Director of Mwanza City and the school principal, which was granted (REF: MCC/SE/20.VOL.II/127). Page 6, 2 ^nd^ paragraph under “ *Ethical approval”* section. 

**Imelda’s** study: We now provided the following details on Imelda’s study (page 6, last paragraph under “ *Ethical approval”* section): *“Imelda’s study received approval from the Makerere University School of Medicine Higher Degrees Research and Ethics Committee (SOMREC; #REC Ref 2018-001) and Uganda National Council for Science and Technology (UNCST; Ref SS4797) to conduct the research on MDSR and community engagement. Engagement of secondary school students in research was preceded by permission from the headmaster and the director of studies. In addition, approval to engage with students in Uganda was obtained from the Ministry of Education and Sports (Uganda). Further, there was a waiver of consent from parents since the research was minimal risk. The students who participated in drama activities and qualitative interviews provided a written consent for those aged 18 years and above, whereas assent was obtained for those aged below 18 years.”* 

**Methods described to carry out the engagements for the case studies:**

- The section on the settings and background to the research projects can be shortened to emphasize important aspects related to the public/community engagements. For example in the Kenya study the authors state a cross-sectional study design and convenient sampling of camel keds. How does this impact on the case studies for community engagement? What dates were these samples collected?

We fully agree and have now rewritten the background information to respond to the constructive comments raised by both reviewers to put the emphasis on community engagement, which is the key subject of this article. Refer to pages 4 – 5 of the revised article under the subheading titled *“The settings and the background to the research project.”* Whilst rewriting this section, we deleted the following sentence: *“…cross-sectional study design and convenient sampling of camel keds”.* 

- What were the ages of the school children in the community engagements?

**Joel’s** study: We now provide the ages of the student volunteers (1 ^st^ paragraph under Ethical approval section) as stated below: *“Oral assent was sought from the student volunteers (aged between 15 – 20 years, mean age = 17.32 years)…” *Page 5, 1 ^st^ paragraph under “ *Ethical approval.”* 

**Denna’s **study: The secondary school students who participated in the consultative meetings, drama and debates activities were high school students and were aged 18 years and above. Page 6, 2 ^nd^ paragraph under “ *Ethical approval”* section.

**Imelda’s **study **:** Age range of the secondary school students was 16 – 21 years, with a mean age of 17.7 years. The students were keen to have their project completed before their final exams, which had been shifted to April 2021. Page 6, last paragraph under “ *Ethical approval”* section.

- A clear distinction should be made for all three case studies as to which aspects were public engagement and which were community engagement.

We thank the reviewer for this constructive comment to distinguish between community engagement *versus* public engagement activities in research for each case study reported here. We define these two terms as follows (see page 3, third paragraph of *“Introduction”* in the revised article): *“We draw on the definition of public engagement used by Cohen et al., (2008: 2): ‘a process that provides people with trustworthy information on key policy issues, elicits their input, and integrates it into decision-making and social action’. They make a distinction between this broader engagement agenda and that of ‘community engagement’, where the people directly participating in or affected by a research project are the focus of engagement. Both are important, and we would argue that as an introduction to broader ‘public engagement’, local community engagement provides a focus for emerging scientists to hone their skills in explaining research concepts and to work with community members to develop ideas, methods and outputs (MacQueen et al., 2015; Musesengwa et al., 2018; Tembo et al., 2021).”* Therefore, all our studies involved community engagement – students are the community in the school. Further, under the sub-section *“What did we learn from the community engagement that we did in THRiVE?*” we state: *“Together, our findings from the community engagement provide key information that could inform policy.”* See page 9.

- The authors state they "designed a study” to assess perceptions of parents and students in Kenya (page 5) and then “developed” an "engagement intervention” referring to an “intervention activity” on page 6.

We now hope that the additional information describing the design of the case study should enable replication in other laboratories. For details on the revisions that address the reviewer’s comments, please refer to the following three subsections article on pages 6 – 9 of the revised article:

- *“Where did the idea for the community engagement focus come from?”*
- *“What the community engagement activity was”, *and
- *“What did we learn from the community engagement that we did in THRiVE?”*

- The authors also “worked within a school setting” to assess awareness of maternal deaths on page 6.

We have provided details of the study activities undertaken in the school. Please refer to page 8 of the revised paper under the subheading titled *“What the community engagement activity was”* 

- Sufficient detail should be given on the processes and methods for community and/or public engagement in the three country settings to be able to replicate such case studies.

We have now provided more procedural details on research processes and methods, community engagement activities, and how the ideas for research engagement developed, to enable replication of the case studies by other laboratories. Please refer to subsection titled *“what the community engagement activity was*” – on pages 6 – 8 of the revised article for details on each case study. In addition, we provide links to two videos that summarize **Joel’s** study on camel health and community engagement ( https://www.youtube.com/watch?v=FuM-RUnjpwM), and access to education by the children from nomadic pastoralist communities ( https://vimeo.com/531249510). Refer to page 6 under the subheading titled *“Where did the idea for the community engagement focus come from?”*

**Reference differing views and opinions:**

- I suggest expanding on ethics in community and public engagement.

**Joel’s** study: We have provided more details on page 5, the 1 ^st^ paragraph under “ *Ethical approval.”* We also state an ethical dilemma faced by the study, as follows: *“The main ethical challenge that Joel faced in his study was the lack of clear strategies on how to give credit to the many community members who contributed to new study ideas during community engagement meetings.” *Refer to page 5, last sentence of the “ *Ethical approval” *section. 

**Denna’s** study: We have provided more information on ethics. The approval granted covered community engagement activities, whereas student engagement in Tanzania required a separate approval from the director of Mwanza City Council (REF: MCC/SE/20.VOL.II/127), in addition to an informed individual consent process prior to research engagement. Please refer to page 6, 2 ^nd^ paragraph under “ *Ethical approval”* section.

**Imelda’s** study: We have provided sufficient details on ethics in community engagement Page 6, last paragraph under “ *Ethical approval”* section.

- I suggest linking the co-development of ideas, research methods and outputs to a theoretical framework such as the co-design approach alluded to in the abstract.

The context of usage of the phrase “co-develop ideas” in the abstract section of our paper refers to new research ideas arising from community engagement aimed at improving relevance of the study. The two-way mutual engagement described in our article was between each individual researcher (THRiVE fellow) and the community members. Thus, there was no research engagement between the three independent case studies led by the early career researchers. In Joel’s study, for instance, mutual engagement and consultation with the camel farmers resulted in co-development of ideas that shaped his work on livestock health as follows: (1) In addition to our initial focus on camel trypanosomiasis and its transmission, another fatal camel disease known as acute camel disease syndrome (ACDS) was reported by farmers during engagement activities. This information led to establishment of a collaborative research study that aimed at understanding the cause and transmission of ACDS for disease control (Getange et al., 2021; Marisol et al., 2022), and (2) we co-identified some insecticidal and repellent plants with potential use in controlling biting keds. This has opened another avenue for Joel’s research on control of keds using plant-derived products. Therefore, our approach to co-design was practical, evolving in each site as we learnt what would be appropriate as engagement activities. We did not have a guiding theoretical framework.  

**Lessons learnt and recommendations:**

- The authors differentiate community engagement from public engagement in the introduction. The authors refer to two-way public engagement on page 7 and community engagement in other sections. Aspects of community and public engagement should be clearly described and/or differentiated if applicable, in the three case studies.

On reflection, we have opted to refer to all our activities as community engagement; all the activities we describe were with local schools. The school students, teachers and parents were a part of the local communities. 

- Are study participants mentioned in the recommendations on page 7, part of the community or public engagements?

Engagements with study participants in our case studies qualify as ‘community engagement’, but not ‘public engagement’. 

- I suggest highlighting the ethical considerations for such engagements.

We have described details on ethics in community engagement (Please refer to the *“Ethical approval”* section on pages 5 – 6 of the revised article). 

- How can the community engagement be scaled up to broader public engagement in resource-challenged and pandemic settings?

We thank the reviewer for this comment. We have added the following information under bullet #4 of the *“Recommendations”* on page 9: *“Communities and the wider public with increased knowledge about ongoing research may be empowered to inform others. Community members who share information gained through their engagement in research can aid reaching out to the general public for example in disseminating research findings to a wider audience, especially to those under resource-challenged settings. Other potential opportunities for efficient scaling up of engagement activities, for instance during pandemics (i.e. COVID-19) to include using social media platforms such as Facebook, WhatsApp, and Twitter as most community members in these studies have access to mobile phones, and also using the mass media (e.g. newspaper articles, opinion pieces, discussions on radio and television).”*

# Reviewer report: Reviewer response for version 1

**DOI:** 10.21956/aasopenres.14494.r29175

**Published:** 2022-04-11

**Authors:** Doreen Tembo

**Affiliations:** 1 Wessex Institute, Faculty of Medicine, University of Southampton, Southampton, UK

The article provides useful learning from three community engagement case studies of early/mid-career researchers working in three different biomedical/applied health focused research areas and methodologies in low and middle income country (LMIC) settings. The case studies utilised a mix of engagement methods ranging from consultation to co-production. They conclude that two-way engagement is crucial in research and that it helps to generate ideas to improve research, strengthen mutual trust between communities and researchers and promote uptake of research findings.

 The open letter will be a useful contribution to the field, especially as there is less published that applied to LMIC countries. The authors are encouraged to consider the following to improve the article:

- Community engagement and its importance is highlighted through the modern lens of Covid/disinformation and distrust at the outset of the article which is great for catching the attention of the reader. However the article should highlight that this was gaining traction before Covid, and that it has a long history within the space of international development. The authors reference refreshed/updated UK National Institute for Health Research (which is referenced incorrectly in the article as ‘National Institutes of Health Research’) INVOLVE’s guidance/briefing notes as support for community engagement within the funding landscape being more novel. However it is not novel in the UK context. The original version of the guidance for example is from 2000. Bioethics which include community engagement have embeded in other funders work (see MacQueen *et al.*, 2015 ^1^).
- The authors are advised to name the case studies (either by the researcher name or a number) to better ensure clarity.
- The researchers involved in the case studies can have their names written in full, first and surname, thereafter they can be referred to consistently by the first or surname, but it would be preferable to choose one.
- It is suggested that the context setting for the case studies is carried out before ethics are discussed to minimise repetition.
- There is potential for the case study scientific background to be shortened to sharing only the key information necessary to understand the community engagement aspects of the paper. For example the length and content of the second case study by Denna achieves this purpose. They should ideally also cover the same elements as they currently report on some different elements, e.g. Imelda’s case study has information on stakeholder engagement while the others appear not to. It would be useful for each case study to have the same subheadings so they can be structured in the same way.
- The language requires a little review to change it from colloquially spoken to written format e.g. "it is not clear on how" should be "it is not clear how", there are some unnecessary capitalisations of words and missing punctuation in other places. Some sentences need to be broken down, for example some are a whole paragraph long.
- The article would benefit from a longer and more critical discussion that draws on wider literature to discuss cross learning across the case studies. It should also critically reflect on any limitations and things which could have been done differently.
- The recommendations add greatly to the value of the paper.
- The following are suggested additional citations or material the authors may wish to consider:
  - https://www.unicef.org/mena/reports/community-engagement-standards
  - https://www.nihr.ac.uk/documents/resource-guide-for-community-engagement-and-involvement-in-global-health-research/27077
  - Musesengwa *et al.* (2018 ^2^).
  - Tembo *et al. *(2021 ^3^).

Does the article adequately reference differing views and opinions?

Partly

Are all factual statements correct, and are statements and arguments made adequately supported by citations?

Partly

Is the Open Letter written in accessible language?

Partly

Where applicable, are recommendations and next steps explained clearly for others to follow?

Yes

Is the rationale for the Open Letter provided in sufficient detail?

Yes

Reviewer Expertise:

Mixed-methods Research, Community Engagement and Involvement Specialist, Health Policy, Health Systems

I confirm that I have read this submission and believe that I have an appropriate level of expertise to confirm that it is of an acceptable scientific standard, however I have significant reservations, as outlined above.

## References

[1] : Evaluating community engagement in global health research: the need for metrics. *BMC Med Ethics* .2015;16: 10.1186/s12910-015-0033-9 44 10.1186/s12910-015-0033-9 26126899PMC4488111

[2] : A Framework for Community and Stakeholder Engagement: Experiences From a Multicenter Study in Southern Africa. *J Empir Res Hum Res Ethics* .13(4) : 10.1177/1556264618769002 323-332 10.1177/1556264618769002 29701110

[3] : Effective engagement and involvement with community stakeholders in the co-production of global health research. *BMJ* .2021; 10.1136/bmj.n178 10.1136/bmj.n178 PMC787927533593805

# Response

**Published:** 2022-06-21

**Authors:** Joel L. Bargul

**Responses to reviewer's comments** The article provides useful learning from three community engagement case studies of early/midcareer researchers working in three different biomedical/applied health-focused research areas and methodologies in low and middle-income country (LMIC) settings. The case studies utilised a mix of engagement methods ranging from consultation to co-production. They conclude that two-way engagement is crucial in research and that it helps to generate ideas to improve research, strengthen mutual trust between communities and researchers and promote uptake of research findings. The open letter will be a useful contribution to the field, especially as there is less published that applied to LMIC countries. The authors are encouraged to consider the following to improve the article:

- Community engagement and its importance is highlighted through the modern lens of Covid/disinformation and distrust at the outset of the article which is great for catching the attention of the reader. However the article should highlight that this was gaining traction before Covid, and that it has a long history within the space of international development.

We agree and have updated the introduction section (page 3) of the revised article to include the following before we mention guidance: *“Community engagement has a long history within the space of international development* *on which to build” * And on page 4 we have mentioned the effect of the pandemic separately. *“While the studies were affected by the COVID-19 pandemic, the* *work proceeded during the pandemic, albeit at a slower pace due to travel* *restrictions, among other guidelines put in place by the governments to control the spread of the disease (Ministry of Health Uganda, 2020).”*

- The authors reference refreshed/updated UK National Institute for Health Research (which is referenced incorrectly in the article as ‘National Institutes of Health Research’) INVOLVE’s guidance/briefing notes as support for community engagement within the funding landscape being more novel. However it is not novel in the UK context. The original version of the guidance for example is from 2000. Bioethics which include community engagement have embedded in other funders work (see MacQueen et al., 2015 ^1^).

Thank you for this observation. We have modified the wording to remove the suggestion that this is a new development. The ‘s’ has been removed from Institute. See page 3 of the revised article under the *“Introduction”*. 

- The authors are advised to name the case studies (either by the researcher name or a number) to better ensure clarity.

We fully agree with the reviewer and have chosen to name each case study by the researcher’s name as shown by the example below. *The Imelda Namagembe Study “Implementation of maternal death surveillance and response policy: the impact of training and community engagement.” *Page 5 under *“The settings and the background to the research project.”* 

- The researchers involved in the case studies can have their names written in full, first and surname, thereafter they can be referred to consistently by the first or surname, but it would be preferable to choose one.

We agree with the reviewer’s suggestion and have therefore adopted consistent format in the revised article, which begins with writing of full names of the researcher, and subsequently, only the first name is referred. 

- It is suggested that the context setting for the case studies is carried out before ethics are discussed to minimise repetition.

We agree with the reviewer, and have now moved the section on *“Ethical approval”* to follow *“The settings and the background to the research project”* section. Refer to page 4 & 5 of the revised article. 

- There is potential for the case study scientific background to be shortened to sharing only the key information necessary to understand the community engagement aspects of the paper. For example the length and content of the second case study by Denna achieves this purpose. They should ideally also cover the same elements as they currently report on some different elements, e.g. Imelda’s case study has information on stakeholder engagement, while the others appear not to. It would be useful for each case study to have the same subheadings so they can be structured in the same way.

We fully agree with this reviewer’s suggestion and have made the following changes for each case study. We have extensively restructured the background information, particularly for Joel and Imelda’s studies (the reviewer was contented with the 3 ^rd^ case study by Denna), to refocus and emphasize community engagement, which is the main idea of our paper. Refer to pages 4 – 5 of the revised article under the subheading titled *“The settings and the background to the research project.”* 

- The language requires a little review to change it from colloquially spoken to written format e.g. "it is not clear on how" should be "it is not clear how", there are some unnecessary capitalisation of words and missing punctuation in other places. Some sentences need to be broken down, for example some are a whole paragraph long.

We have corrected these errors. We have now carefully addressed to ensure adherence to Standard English throughout the revised article. 

- The article would benefit from a longer and more critical discussion that draws on wider literature to discuss cross learning across the case studies. It should also critically reflect on any limitations and things, which could have been done differently.

We have added more literature by referring to six new sources (references) to widen the scope of the paper. In addition, we have added the following paragraph on the limitations of the study that reflects what we could have done differently (page 9 under *“A limitation of the study”*): *“Our case studies would have benefitted from a more rigorous approach to measuring and evaluating the socio-economic impact of the community engagement activities. However, monitoring and evaluation of the engagement outcomes were not conducted due to the limited funding available.”*

- The recommendations add greatly to the value of the paper.

Thank you very much for this encouraging comment.

- The following are suggested additional citations or material the authors may wish to consider:

References:

1. MacQueen KM, Bhan A, Frohlich J, Holzer J, et al.: Evaluating community engagement in global health research: the need for metrics.BMC Med Ethics. 2015; 16: 44. DOI: 10.1186/s12910-015-0033-9.
2. Musesengwa R, Chimbari MJ, Mukaratirwa S: A Framework for Community and Stakeholder Engagement: Experiences From a Multicenter Study in Southern Africa (2018). J Empir Res Hum Res Ethics. 13 (4): 323-332. DOI: 10.1177/1556264618769002
3. Tembo D, Hickey G, Montenegro C, Chandler D, et al.: Effective engagement and involvement with community stakeholders in the co-production of global health research. BMJ. 2021. https://doi.org/10.1136/bmj.n178.
4. https://www.unicef.org/mena/reports/community-engagement-standards
5. https://www.nihr.ac.uk/documents/resource-guide-for-community-engagement-andinvolvement-in-global-health-research/27077
6. Ministry of Health Uganda. Guidelines for prevention of Covid-19 when conducting meetings at workplaces. Kampala. 2020 https://www.health.go.ug/covid/project/guidelines/.

We thank the reviewer for taking an extra step to suggest these additional references that we have cited in our revised paper. We also have included one more reference, i.e. Ministry of Health Uganda, 2020 (#6 in the above list)
